# Feeding-Artery Microvascular Plug Embolization Versus Nidus-Plus-Feeding-Artery Coil Embolization of Pulmonary Arteriovenous Malformations

**DOI:** 10.3390/jcm14092980

**Published:** 2025-04-25

**Authors:** Shanmukha Srinivas, Dustin G. Roberts, Justin P. McWilliams, Lucas R. Cusumano

**Affiliations:** Division of Interventional Radiology, University of California, Los Angeles, CA 90095, USA; ssrinivas@mednet.ucla.edu (S.S.); jumcwilliams@mednet.ucla.edu (J.P.M.)

**Keywords:** pulmonary arteriovenous malformation, coil embolization, microvascular plug

## Abstract

**Background/Objectives**: Microvascular plug embolization in the distal feeding artery (FA-MVP) and coil embolization targeting the nidus and feeding artery (NiFA-coil) are effective treatments for pulmonary arteriovenous malformations (PAVMs). This study compares their outcomes. **Methods**: A retrospective chart review was conducted on patients who underwent NiFA-coil or FA-MVP embolization for PAVMs between October 2014 and May 2024, with initial (short-term) follow-up chest CT imaging performed within 18 months, and the latest (long-term) follow-up performed at least 3 years post-treatment. Durable occlusion was defined as ≥70% shrinkage of the nidus or draining vein on follow-up CT. A Cox proportional hazards regression model assessed the association between technique and durable occlusion, with inverse propensity score weighting used to adjust for patient and PAVM characteristics. **Results**: A total of 142 PAVMs (48 FA-MVP, 94 NiFA-coil) in 85 patients were analyzed. Durable occlusion was 97.2% (138/142) at a median short-term follow-up of 4.2 months and 90.2% (37/41) at a median long-term follow-up of 56.0 months. Simple PAVMs were more frequent in the FA-MVP group (93.8%, 45/48) than in the NiFA-coil group (61.2%, 58/94) (*p* < 0.001). The NiFA-coil group had larger feeding arteries (3.8 mm vs. 2.3 mm, *p* < 0.001) and sac sizes (13.1 mm vs. 7.7 mm, *p* = 0.040). Short and long-term durable occlusion rates were comparable (NiFA-coil: 96.8% and 88.9%; FA-MVP: 97.8% and 92.9%, respectively; *p* > 0.99, *p* > 0.99). After propensity score matching, compared to FA-MVP, NiFA-coil had a hazard ratio for short-term persistence of 1.06 (95% CI, 0.16–6.99; *p* = 0.956). **Conclusions**: Both NiFA-coil and FA-MVP embolization are highly effective for PAVM treatment, with similar success rates.

## 1. Introduction

Arteriovenous malformations (AVMs) are a subset of congenital vascular malformations with distorted angioarchitecture resulting in abnormal connections between arteries and veins with no intervening capillary bed [1]. AVMs with simple angioarchitecture have a single feeding artery (FA), while AVMs with a complex angioarchitecture have two or more FAs. Pulmonary AVMs (PAVMs) are a type of AVM found in the lung and commonly occur in patients with genetic conditions such as Hereditary Hemorrhagic Telangiectasia (HHT) [1]. These aberrant communications result in right-to-left shunting, bypassing the oxygenation and filtration performed in the capillary bed [2]. This can lead to complications, including hypoxemia, paradoxical emboli, and neurological manifestations such as brain abscess and stroke [2]. Endovascular embolization has become a cornerstone in managing PAVMs.

Coils were one of the first endovascular embolic agents used to treat PAVMs and function by mechanical occlusion via the formation of a dense cross-sectional framework and/or thrombotic occlusion via platelet-driven clot formation [3,4,5,6]. Coil designs have evolved over time, including the development of hydrogel-impregnated coils and high-volume detachable non-fibered coils, which both have shown lower persistence rates compared to traditional coils [7,8]. While coils have historically been deployed within the distal feeding artery (FA) alone, several studies have demonstrated improved durable occlusion using nidus-plus-feeding artery (NiFA) coil embolization [9,10,11,12].

The MicroVascular Plug (MVP) (Medtronic; Minneapolis, Minnesota) is a more recently developed endovascular occlusion device comprised of a self-expandable nitinol cage covered by a polytetrafluoroethylene membrane (PTFE) and is advantageous for its short landing zone of deployment and rapid mechanical occlusion [13]. Several studies have demonstrated durable occlusion rates of over 94% for feeding artery (FA) MVP embolization [14,15,16,17]. Comparative studies have demonstrated higher durable occlusion rates of FA-MVP embolization compared to coil embolization alone [14,15,18,19,20]. However, these comparative studies used the feeding-artery-only coil embolization technique, and comparative studies of NiFA-coil embolization versus FA-MVP embolization have not been performed.

This retrospective study aims to compare durable occlusion rates between NiFA-coil embolization and distal FA-MVP embolization while using propensity score weighting to account for biases in technique selection.

## 2. Materials and Methods

### 2.1. Study Population

**Figure 1:**
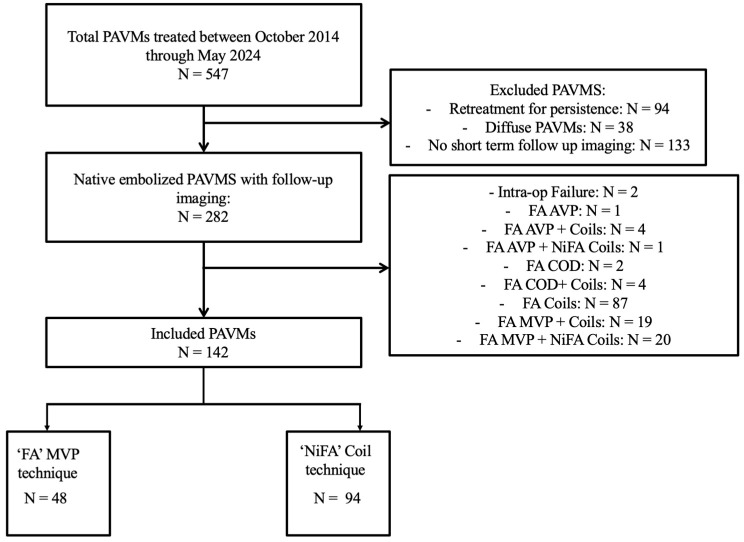
Flowchart depicting inclusion and exclusion criteria. PAVM = pulmonary arteriovenous malformation; FA = distal feeding artery technique; NiFA = nidus-plus-feeding-artery technique; AVP = Amplatzer Vascular Plug; COD = Caterpillar Occlusion Device.

Approval from the Institutional Review Board (IRB) was obtained with a waiver of informed consent to conduct a HIPAA-compliant retrospective chart review of patients with pulmonary arteriovenous malformations (PAVMs) who underwent embolization between October 2014 and May 2024 at a single Hereditary Hemorrhagic Telangiectasia (HHT) Center of Excellence. Data on patient age, sex, HHT status, and available genetic data were collected and analyzed. Exclusion criteria for treated PAVMs included the following: absence of follow-up computed tomography (CT) imaging between 1 and 18 months after the procedure to ensure uniform short-term follow-up intervals; evidence of diffuse disease, indicated by PAVMs present in all subsegmental branches of a minimum of one lung segment; embolization of recurrent or residual PAVMs previously treated; intraprocedural technical failure, defined as the inability to achieve complete angiographic occlusion absence of contrast enhancement in the nidus or outflow vein at the time of the procedure; use of the Amplatzer Vascular Plug (AVP) (Abbott Cardiovascular; St. Paul, MN, USA); use of the Caterpillar Occlusion Device (COD) (Becton Dickinson; Franklin Lakes, NJ, USA); coil embolization of the feeding-artery-only; and combined use of MVPs and coils for the same PAVM. From an initial pool of 547 PAVMs, 142 newly diagnosed PAVMs in 85 patients treated over 92 sessions were included in the final analysis. (Figure 1).

The average age at the time of treatment was 46.5 years (range, 9–77), with 58 patients (68.2%) being female. HHT was confirmed in 68 patients (80.0%) diagnosed clinically when at least three of the four Curacao criteria were met [20]. Genetic testing performed on 39 patients identified 26 individuals with ENG mutations (HHT Type 1), 6 with ACVRL1 mutations (HHT Type 2), 1 with a SMAD4 mutation, and 6 with no identifiable genetic mutations. There were no significant differences in patient demographics across the different embolization techniques (Table 1).

### 2.2. PAVM Features and Treatment Approach

PAVM characteristics, including location, laterality, angioarchitecture, FA diameter, and nidal sac diameter, were assessed based on the review of pretreatment CT scans and intraprocedural fluoroscopic images. This classification was carried out by an interventional radiology resident and validated by an attending interventional radiology physician. PAVMs with a single feeding artery (FA) were classified as having simple angioarchitecture, while those with two or more FAs were classified as having complex angioarchitecture [21]. PAVMs were further categorized as “saccular” or “nonsaccular” based on whether the nidus exhibited aneurysmal dilation, defined as a sac diameter more than twice the diameter of the largest FA. Sac diameters were measured at their largest trans-axial dimension.

Procedures were performed by two staff interventional radiologists with 2 and 16 years of experience. CT imaging obtained prior to the procedure, with or without contrast enhancement, was available for all patients and used to guide treatment planning.

### 2.3. Embolization Technique

All procedures were performed under moderate intravenous sedation on an outpatient basis, utilizing right common femoral venous access. A 6F sheath or 7F guide catheter was positioned in the main pulmonary artery. Patients received an initial heparin bolus of 3000–5000 units, followed by 1000 units per hour. Digital subtraction pulmonary angiography was performed using a 5F pigtail catheter in a contralateral oblique projection to evaluate PAVM size and anatomy. The feeding artery was selected using a 5F angled hydrophilic angiographic catheter, while superselection of the distal feeding artery or nidus, when necessary, was achieved with microcatheters ranging from 1.9F to 2.8F, advanced over a 0.014″ microwire.

**Figure 2:**
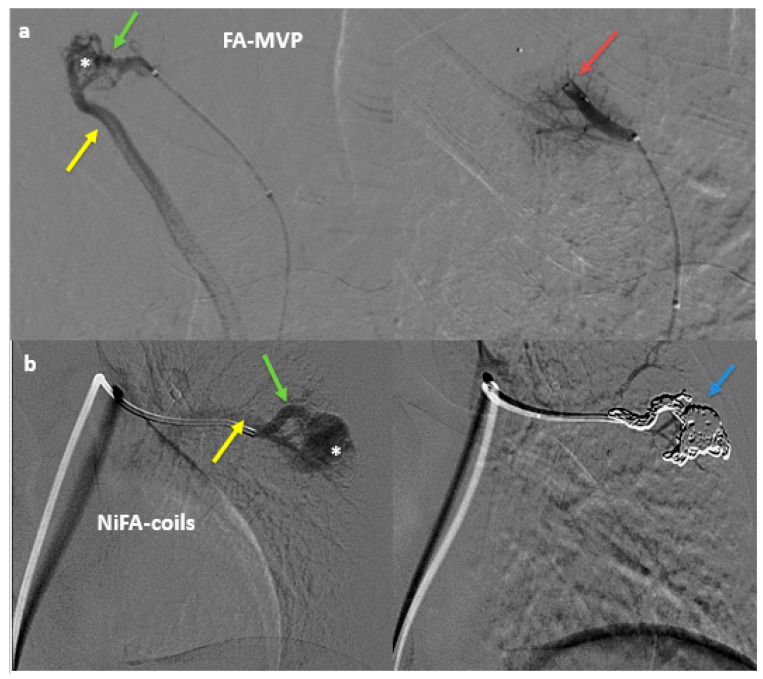
Digital subtraction angiography (DSA) before (left) and after (right) embolization of (**a**) feeding artery (FA) (green arrow) microvascular plug (MVP) (red arrow) embolization in a right upper lobe pulmonary arteriovenous malformation (PAVM) with a nidus (asterisk). (**b**) DSA before (left) and after (right) coil embolization of the dominant feeding artery (green arrow) and nidus (asterisk) using two Ruby 8 mm × 60 cm coils and one 60 cm Packing coil (blue arrow) in a left lower lobe PAVM. Draining veins (yellow arrow) are seen only prior to embolization.

For FA-MVP embolization, a single MVP was deployed within the distal feeding artery (within 1 cm of the nidus), followed rarely by a second MVP to achieve complete angiographic stasis. NiFA-coil embolization was performed by densely packing the nidus or sac with coils and extending the coil pack 1–2 cm into the distal feeding artery (Figure 2). Coil selection was guided by angiographic findings, with coil sizing matched to vessel diameter at a 1:1 ratio. The coil embolics deployed were AZUR and AZUR CX (Terumo Medical; Tokyo, Japan), Interlock (Boston Scientific; Marlborough, MA, USA), Nester and Tornado (Cook Medical; Bloomington, IN, USA), Concerto (Medtronic), and Ruby (Penumbra; Alameda, CA, USA) (Table 2). Ruby coils are high-volume, detachable, non-fibered (HVDNF) coils, while Azur coils are detachable, non-fibered hydrogel (DNFH) coils. Pulmonary arteriovenous malformations (PAVMs) were categorized into two groups: those treated exclusively with traditional non-HVDNF/DNFH coils and those treated with HVDNF/DNFH coils, either alone or in combination with other coils.

The procedural endpoint of embolization was complete angiographic occlusion, confirmed by the absence of contrast opacification in the nidus or draining vein for at least 5 heartbeats. When using expansion-dependent coils, operators waited five minutes to ensure complete occlusion before performing the final angiographic assessment.

### 2.4. Follow-Up

Patients were typically discharged on the same day, with a single case requiring overnight admission for pain and nausea management. Complications were documented according to the Society of Interventional Radiology’s adverse event classification system. For pain management, patients were advised to use over-the-counter acetaminophen or nonsteroidal anti-inflammatory drugs as needed. A follow-up chest CT with or without contrast was performed at least one month after embolization, at which point patients returned to the interventional radiology clinic for an evaluation of symptoms and treatment response. Post-procedural imaging was initially reviewed by an interventional radiology resident and subsequently assessed independently by an attending interventional radiologist.

Routine surveillance chest CT imaging was conducted every 3–5 years or sooner if new symptoms arose. The earliest available CT scan between 1 and 18 months post-procedure was used to evaluate short-term treatment effectiveness, while the most recent scan beyond three years, when available, was used to assess long-term success. Consistent with existing literature, a successful treatment response was defined as a reduction in PAVM sac or draining vein size by at least 70%, which was manually calculated from pre- and post-embolization imaging [5].

### 2.5. Statistical Analysis

Statistical analysis was conducted using RStudio version 1.3.1056 (R Foundation for Statistical Computing, Vienna, Austria). Bivariate analyses were used to compare baseline characteristics across embolization techniques and between successful and unsuccessful treatments. The Fisher exact test and Mann–Whitney U test were applied to assess differences in categorical and continuous variables, respectively.

To mitigate biases related to embolization technique selection, additional propensity score weighting analyses were conducted to balance covariates between PAVMs treated with each approach. Associations between treatment type and various patient or PAVM characteristics—including age, sex, HHT status, largest FA diameter, PAVM location, presence of a sac, angioarchitecture, and timing of the 1- to 18-month follow-up—were evaluated. The Shapiro–Wilk test was applied to assess normality in continuous variables, while the Kruskal–Wallis or Fisher exact test was used to analyze associations between devices and these variables. Variables with a *p*-value < 0.25 in these initial comparisons were included in estimating propensity score weights for the model, which was then used to assess associations between embolic device type and PAVM persistence.

A Cox proportional hazards regression model was employed to evaluate the relationship between embolic device selection and time to PAVM persistence during short-term follow-up, accounting for the duration of post-embolization monitoring for each patient. Inverse propensity score weighting was employed to account for selection bias in embolic device use based on patient and PAVM characteristics. The covariate balance between PAVMs treated with different techniques was evaluated both before and after weighting. Variables with residual imbalance following weighting (defined by a weighted standardized difference ≥ 0.2) were included as additional covariates in the regression model for further adjustment.

## 3. Results

### 3.1. PAVM Characteristics

A total of 142 previously untreated PAVMs underwent embolization, of which 103 (72.5%) were simple, and 39 (27.5%) were complex. PAVMs most frequently were located in the lower lobes, with 25.4% in the left lower lobe and 23.2% in the right lower lobe. The average feeding artery (FA) diameter was 3.3 mm (range: 1.5–11 mm), with 92 (64.8%) of the treated PAVMs measuring 3 mm or less. “Saccular” morphology was observed in 95 PAVMs (66.9%), with a mean sac diameter of 11.2 mm (range: 3.3–46.0 mm).

Complex PAVMs had significantly larger FAs (mean dominant FA diameter: 4.3 mm) compared to simple PAVMs (mean FA diameter: 2.9 mm) (*p* < 0.001), while sac size was also larger in complex PAVMs (mean diameter: 14.2 mm vs. 10.5 mm), though this difference was not statistically significant (*p* = 0.111). PAVMs treated with NiFA-coil embolization were more likely to be complex (*p* < 0.001), have a larger feeding artery (*p* < 0.001), and a larger sac size (*p* < 0.001). Additionally, the NiFA-coil group had a higher percentage of left lower lobe PAVMs (30.9% vs. 14.6%), while the FA-MVP group had a greater proportion of right middle lobe PAVMs (33.3% vs. 11.7%) (*p* = 0.032).

Of note, while fluoroscopy time did not differ between procedures involving NiFA coils and FA-MVP embolization (mean: 33.7 min vs. 32.8 min; *p* = 0.621), NiFA-coil embolization was more expensive per PAVM compared to FA-MVP embolization (mean embolic cost per PAVM: 7027 USD vs. 2599 USD, *p* < 0.001). When stratified by PAVM complexity, the costs for complex PAVMs (mean: 6160 USD) were significantly higher than for simple PAVMs (mean: 4439 USD) (*p* < 0.001). For complex PAVMs, NiFA-coil embolization (mean: 8836 USD) was more expensive than FA-MVP embolization (mean: 3327 USD), and this difference approached statistical significance (*p* = 0.061). For simple PAVMs, NiFA-coil embolization (mean: 5905 USD) remained significantly more expensive than FA-MVP (2550 USD, *p* < 0.001).

Table 3 provides a detailed summary of PAVM characteristics based on the embolization technique used.

### 3.2. Embolization Technique and Outcomes

A total of 138 PAVMs (97.2%) were successfully treated with embolization, with an average short-term follow-up of 5.6 months (median 4.2 months; range 1.0–17.7 months). Long-term follow-up (beyond 3 years) was available for 41 PAVMs, with a mean follow-up of 57.4 months (median 56.0 months; range 37.1–87.7 months). No significant differences were found between the treatment groups regarding the proportion of PAVMs with long-term follow-up (*p* > 0.99), duration of short-term follow-up (*p* = 0.868), or the duration of long-term follow-up (*p* = 0.895). A total of 90 (96.8%) procedures were conducted by a staff interventional radiologist with 16 years of experience, while three (3.2%) procedures were performed by a staff interventional radiologist with 2 years of experience.

Regarding angioarchitecture, simple PAVMs showed higher success rates in both short-term (102/103, 99.0%) and long-term (29/30, 96.7%) compared to complex PAVMs (36/39, 92.3%; 8/11, 72.7%), and the difference approached statistical significance (*p* = 0.063; *p* = 0.052). Simple PAVMs treated with the NiFA-coil technique showed higher success rates in both short-term (58/58, 100%) and long-term (17/17, 100%) follow-up compared to complex PAVMs (33/36, 91.7%; 7/10, 70%) (*p* = 0.054; *p* = 0.041). Only 6% of complex PAVMs were treated with FA-MVP, limiting the ability to compare outcomes between simple and complex PAVMs in the FA-MVP cohort.

**Figure 3:**
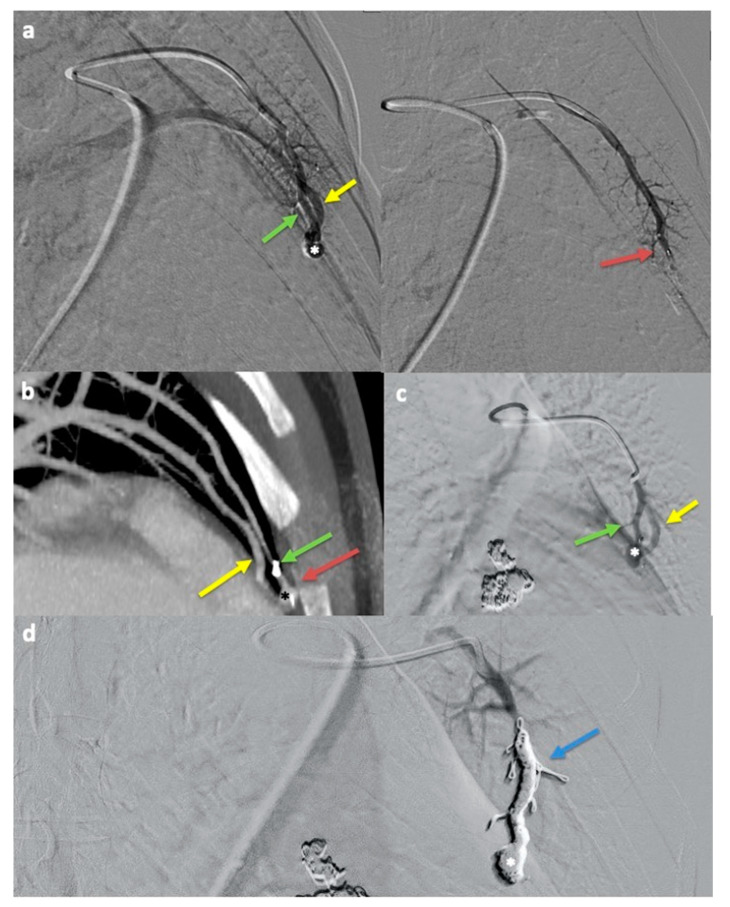
Treatment failure and retreatment after feeding artery (FA) microvascular plug (MVP) embolization. (**a**) Digital subtraction angiography before (left) and after (right) feeding artery (FA) (green arrow) microvascular plug (MVP) (red arrow) embolization of a pulmonary arteriovenous malformation (PAVM) in the lingula. The draining vein (yellow arrow) is seen opacified before but not after embolization. (**b**) Coronal maximum intensity projection (MIP) CT angiography obtained 14 months after the procedure demonstrates residual opacification in the nidus (asterisk) and draining vein (yellow arrow) distal to the MVP (red arrow) and FA (green arrow), consistent with treatment failure due to recanalization. (**c**) Digital subtraction angiography 5 years after the initial procedure demonstrates recanalization of the nidus (asterisk) and draining vein (yellow arrow) distal to the FA (green arrow). (**d**) Following coil embolization of the dominant feeding artery and nidus using a Ruby 6 mm × 30 cm soft coil and two 60 cm packing coils (blue arrow), the draining vein and nidus (asterisk) no longer opacify.

**Figure 4:**
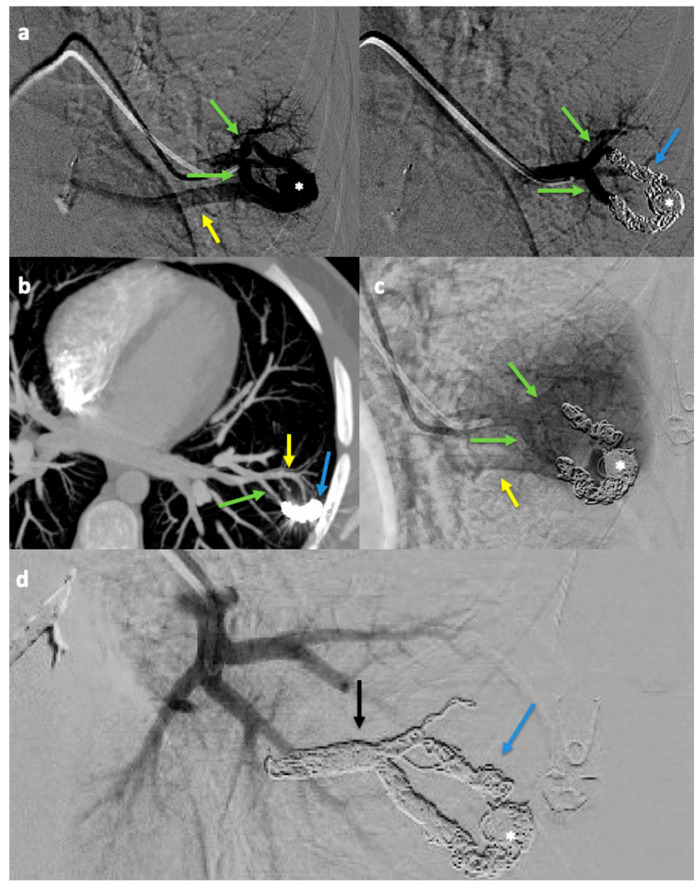
Treatment failure and retreatment after nidus-plus-feeding artery (NiFA) coil embolization (**a**) Digital subtraction angiography (DSA) before (left) and after (right) coil embolization of the dominant feeding arteries (green arrows) and nidus (asterisk) using Interlock-18 standard 8 mm × 20 cm, three 6 mm × 20 cm, one 4 mm × 12 cm, one 3 mm × 12 cm, one interlock-18 soft 4 mm × 12 cm, and three 3 mm × 14 cm microNester coils (blue arrow) in a left lower lobe PAVM. The draining vein (yellow arrow) is seen only prior to embolization. (**b**) Axial maximum intensity projection (MIP) CT angiography obtained 14 months after the procedure demonstrates residual flow through the coil pack (blue arrow) and draining vein (yellow arrow) distal to a dominant FA (green arrow), consistent with treatment failure due to recanalization. (**c**) Digital subtraction angiography 5 years after the initial procedure demonstrates recanalization of the nidus (asterisk) and draining vein (yellow arrow) distal to the dominant feeding arteries (green arrows). (**d**) Following coil embolization of the two dominant feeding arteries proximal to the pre-existing coil pack (blue arrow) using a Ruby 3 mm × 15 cm standard coil and two Ruby 60 cm packing coils (black arrow), the draining vein and nidus (asterisk) no longer opacify.

Among all 142 PAVMs, 48 (33.8%) were treated with the FA-MVP technique, and 94 (66.2%) were treated with the NiFA-coil technique. The short-term (91/94, 96.8%) and long-term (24/27, 88.9%) success rates for the NiFA-coil technique were not significantly different from those of the FA-MVP technique (47/48, 97.9%; 13/14, 92.9%) (*p* > 0.99 for both). Stratified analysis by PAVM feature revealed significant differences between the two techniques in feeding artery diameter (*p* < 0.001 for short-term; *p* = 0.004 for long-term) and sac diameter (*p* < 0.001 for short-term; *p* = 0.004 for long-term) in successful cases. Treatment failure after FA-MVP embolization is demonstrated in Figure 3. Treatment failure after NiFA-coil embolization is demonstrated in Figure 4.

Bivariate analysis showed no significant differences in persistence rates between small (≤3 mm) and large (>3 mm) feeding artery diameters in either short-term or long-term follow-up. Success rates for small FAs were 90/92 (97.8%) for short-term and 23/25 (92%) for long-term, while large FAs had success rates of 48/50 (96.0%) for short-term and 14/16 (87.5%) for long-term (*p* = 0.613 for short-term; *p* = 0.637 for long-term). Additionally, the presence of “saccular” morphology was not predictive of success in either short or long-term follow-up: 93/95 (97.9%) and 21/22 (95.4%) for saccular PAVMs versus 45/47 (95.7%) and 16/19 (84.2%) for nonsaccular PAVMs (*p* = 0.600 for short-term; *p* = 0.321 for long-term).

Stratified treatment outcomes with subgroup analyses comparing FA-MVP and NiFA-coil techniques are detailed for short-term follow-up in Table 4 and Table 5.

Additional subgroup analysis of PAVMs treated with NiFA coil embolization showed no significant differences in baseline demographics or durable occlusion rates between the two groups: 27 PAVMs (28.7%) treated exclusively with traditional non-HVDNF/DNFH coils and 67 PAVMs (71.3%) treated with HVDNF/DNFH coils, either alone or in combination with other coils (Table 6). Notably, traditional coil embolization required more coils than HVDNF/DNFH embolization (*p* < 0.001).

### 3.3. Model for Detecting Association Between Treatment Type and Pulmonary Arteriovenous Malformation

Persistent differences between the treatment groups at *p* < 0.25 were observed for the largest feeding artery diameter, PAVM location, and PAVM angioarchitecture. As a result, these variables were incorporated into the propensity score weighting process. The standardized differences for these variables among PAVMs treated with the two different treatment types were assessed both before and after applying the weighting (Table 7). Feeding artery diameter remained unbalanced with a weighted standardized difference >0.2 and was therefore included in the Cox regression model. The mean feeding artery diameter was 3.8 ± 1.9 mm for NiFA-coils and 2.3 ± 0.7 mm for FA-MVP (*p* < 0.001).

Table 8 represents the Cox regression model, which includes inverse propensity score weighting and an adjustment for feeding artery diameter due to the remaining imbalance in feeding artery diameter after weighting. FA-MVP was set as the referent. Relative to FA-MVP, NiFA-coils had a hazard ratio for persistence of 1.06 (95% CI, 0.16 to 6.99; *p* = 0.956). In the model, following adjustment for treatment type, feeding artery diameter was not found to be significantly associated with persistence (hazard ratio, 1.24; 95% CI, 0–434.97; *p* = 0.943).

### 3.4. Adverse Events

Fifteen minor complications (16.3%) were reported across 92 procedures, as per the Society of Interventional Radiology criteria. No incidents of coil migration or PAVM rupture were noted. The most frequently observed complication was mild pleuritic chest pain, which most often was self-limited or resolved with a short course of nonsteroidal anti-inflammatory drugs. This affected 10 (10.9%) patients and 13 treated PAVMs. No link was found between the complication rates and the embolization technique used.

Other less common complications included 1 (1.1%) case of redness and swelling at the access site (likely cellulitis), which improved with antibiotics; 1 (1.1%) access site hematoma that was managed with manual compression; 2 (2.2%) mild and self-limited nausea episodes for which one patient elected to be admitted overnight for observation; 2 (2.0%) patients who needed antibiotics for suspected pneumonia; and 1 (1.1%) episode of self-limited hemoptysis, which resolved within a week in a patient who was also receiving bronchial artery embolization for pre-procedure hemoptysis.

Additionally, one patient experienced coffee ground emesis, likely unrelated to the procedure, which resolved spontaneously while recovering in the post-procedure unit. This patient chose to forgo overnight observation and reported no further symptoms during a follow-up visit.

## 4. Discussion

This study found no significant differences in the durable occlusion rates between NiFA-coil embolization and FA-MVP embolization for the treatment of PAVMs. The short-term occlusion rate for the NiFA-coil group was 97.2%, and the long-term occlusion rate was 88.9%, while the FA-MVP group had a short-term occlusion rate of 97.9% and a long-term occlusion rate of 92.9% (*p* > 0.99 for both). Key factors influencing treatment outcomes included PAVM angioarchitecture, with complex PAVMs having significantly larger feeding arteries and sac diameters. These complex PAVMs were more likely to experience both short- and long-term recurrence compared to simple PAVMs. Notably, PAVMs treated with NiFA-coils were more likely to be complex and to have larger feeding arteries and sacs when compared to those treated with FA-MVP. Incidentally, PAVMs treated with NiFA-coils were more often located in the left lower lobe, while FA-MVP-treated PAVMs were more frequently located in the right middle lobe. Despite these anatomical differences, the durable occlusion rates did not differ significantly between the techniques after stratification by PAVM characteristics and propensity score matching. These are both viable treatment options for PAVMs in patients with diseases such as HHT, which was present in 80% of the patients in this study.

While this study supports previous research indicating that various factors such as pulmonary hypertension, PAVM angioarchitecture, and feeding artery size may influence durable occlusion, it also demonstrates that anatomical features can guide the selection of embolization technique [19,22]. The high success rate of NiFA-coils—which was non-inferior to FA-MVP despite a higher proportion of complex PAVMs—challenges prior studies suggesting that MVP embolization has superior outcomes compared to coil embolization for PAVMs [14,15,18,19,20] although these studies utilized feeding-artery-only coil embolization. In complex PAVMs, the number, size, and tortuosity of feeding arteries may increase the difficulty of embolic deployment in each feeding artery. If these feeding arteries cannot be treated with an MVP due to these factors, embolization of the PAVM nidus with coils may prevent persistence. Furthermore, the increased number of coils, which is more feasible with the NiFA technique compared to feeding-artery-only coil embolization, may help explain the low recurrence risk observed in this study [23]. The majority of PAVMs undergoing NiFA coil embolization (71.3%) were treated using HVDNF or DNFH coils. These coils allowed for higher packing density and required fewer coils per PAVM compared to non-HVDNF/DNFH coils. Notably, there was no significant difference in durable occlusion rates between HVDNF/DNFH and non-HVDNF/DNFH coils, suggesting that the NiFA technique was a more significant factor contributing to high occlusion rates than the specific coil technology used.

A notable cost difference was found between treatment groups. NiFA-coil embolization was significantly more expensive (mean cost: 7027 USD vs. 2599 USD for FA-MVP), and this relationship held true for both simple and complex PAVMs. This aligns with prior research showing similar technical success rates between FA-MVP and coil embolization but highlighting the advantages of FA-MVP in terms of lower procedure cost and reduced time to occlusion [23]. In clinical practice, FA-MVP is likely a more cost-effective option, particularly when patient and PAVM characteristics are comparable. However, NiFA-coils may still be preferred in cases with more complex PAVMs with multiple feeding arteries or PAVMs with larger feeding artery sizes.

The complication rate in this study was low, with the most common adverse event being mild pleuritic chest pain, which typically resolved on its own or with short-term medical treatment. Other minor complications included access site hematomas, nausea, and mild respiratory issues. Notably, no major adverse events, such as coil migration, PAVM rupture, or any neurological sequelae, were reported, confirming the safety of both embolization techniques. The absence of a clear link between complication rates and embolization technique further suggests that both NiFA-coils and FA-MVP have similar safety profiles.

Several limitations of this study should be noted. Durable occlusion was defined using a threshold of ≥70% nidus or draining vein shrinkage as a marker of durable occlusion on CT chest imaging. Although this standard is widely used, it has some limitations for predicting PAVM recanalization as it does not necessarily equate to complete cessation of blood flow through the PAVM [24]. Baseline differences in PAVM location were observed, but these were adjusted for in the propensity-matched analysis. There was a small number of complex PAVMs in the FA-MVP group, which limited sub-analysis of simple versus complex PAVM durable occlusion with FA-MVP embolization. Furthermore, while various coil types were used in this study, the comparison between techniques was limited to coils commonly used after 2014, which may affect the generalizability of results when compared to older coil technology [25]. Most procedures were performed by a single operator with over 15 years of experience at an HHT center of excellence, which may limit the generalizability of these findings to centers with less experience or to operators outside of HHT centers of excellence. Lastly, this study did not compare the two embolization techniques to other vascular plugs, such as the Amplatzer vascular plug, which has also shown high technical success rates in previous research [26,27,28].

## 5. Conclusions

Both NiFA-coil and FA-MVP embolization techniques are highly effective and safe for the treatment of PAVMs. While NiFA-coil embolization was more commonly used for complex PAVMs with larger feeding arteries and sacs, both techniques achieved comparable success rates in short- and long-term follow-ups. The cost was lower for FA-MVP embolization.

## Figures and Tables

**Table 1:** Patient characteristics.

Patient Characteristics	All Patients (n = 85) *	NiFA Coil (n = 63)	FA-MVP (n = 36)	*p*-Value
Age (years)	46.5 ± 17.4	45.3 ± 17.5	47.1 ± 17.6	0.593
Gender				
Male	27 (31.8%)	21 (33.3%)	10 (27.8%)	0.655
Female	58 (68.2%)	42 (66.7%)	26 (72.2%)	
HHT Status				
Negative	17 (20.0%)	12 (19.0%)	5 (13.9%)	0.396
Positive	68 (80.0%)	51 (81.0%)	31 (86.1%)	
Genetic Diagnosis (n = 33)				
ENG	26 (78.8%)	20 (87.0%)	11 (73.3%)	0.626
ACVRL1	6 (18.2%)	2 (8.7%)	4 (26.7%)	
SMAD4	1 (3.0%)	1 (4.3%)	0 (0%)	

*p*-values from Fisher’s exact test for categorical variables. FA, feeding artery; MVP, MicroVascular Plug; NiFA, nidus-plus-feeding-artery; HHT, hereditary hemorrhagic telangiectasia. * 13 patients with multiple PAVMs were embolized using both techniques.

**Table 2:** Coil Embolic Data.

Embolic Brand	Coil Category	Manufacturer		Total
	Detachable Fibered	Boston Scientific, Marlborough, MA, USA		
Inter-lock		Interlock 018	147	234
		Interlock 018 soft	27	
		Interlock 035	60	
Tornado	Pushable Fibered	Cook Medical, Bloomington, IN, USA		15
	High-Volume Detachable Non-Fibered (HVDNF)	Penumbra, Alameda, CA, USA		
Ruby		Ruby Standard Coil	85	233
		Ruby Soft Coil	64	
		Packing Coil	76	
		POD Coil	8	
Nester	Pushable Fibered	Cook Medical, Bloomington, IN, USA		78
Micro Nester	Pushable Fibered	Cook Medical, Bloomington, IN, USA		21
Azur	Detachable Non-Fibered Hydrogel (DNFH)	Terumo Medical, Somerset, NJ, USA		28
Concerto	Detachable Fibered	Medtronic, Minneapolis, MN, USA		7
				616

**Table 3:** PAVM Characteristics by Embolization Technique.

PAVM Characteristic	All PAVMs (n = 142)	NiFA Coil (n = 94)	FA-MVP (n = 48)	*p*-Value
Angioarchitecture				
Simple	103 (72.5%)	58 (61.7%)	45 (93.8%)	<0.001
Complex	39 (27.5%)	36 (38.3%)	3 (6.2%)	
Nonsaccular	47 (33.1%)	32 (34.0%)	15 (31.3%)	0.851
Saccular	95 (66.9%)	62 (66.0%)	33 (68.9%)	
Size				
FA diameter (mm)	3.3 ± 1.8	3.8 ± 1.9	2.3 ± 0.7	<0.001
Sac diameter (mm) *	11.2 ± 7.8	13.1 ± 8.9	7.7 ± 3.2	<0.001
Location				
Lingula	18 (12.7%)	12 (12.8%)	6 (12.5%)	0.032
LLL	36 (25.4%)	29 (30.9%)	7 (14.6%)	
LUL	14 (9.9%)	10 (10.6%)	4 (8.3%)	
RUL	14 (9.9%)	8 (8.5%)	6 (12.5%)	
RML	27 (19.9%)	11 (11.7%)	16 (33.3%)	
RLL	33 (23.2%)	24 (25.5%)	9 (18.8%)	
Embolics				
No. of coils per PAVM		6.7 ± 5.3		
Price (USD per PAVM)	5530 ± 4724	7027 ± 5197	2599 ± 504	<0.001
Fluoroscopy time (min) †	33.5 ± 13.2	33.7 ± 11.6	32.8 ± 18.5	0.621
Short-term follow-up duration (months per PAVM)	5.6 ± 3.9 4.2 (0.6–17.7)	5.8 ± 4.1 4.2 (1.1–16.5)	5.2 ± 3.5 4.2 (0.6–17.7)	0.868
Long-term (>3 years) follow-up present	41 (28.9%)	27 (28.7%)	14 (29.2%)	1
Long-term (>3 years) follow-up duration ‡ (months per PAVM)	57.4 ± 15.5 56.0 (37.1–87.7)	57.7 ± 15.9 56.0 (37.1–87.7)	55.9 ± 14.9 53.3 (45.9–79.3)	0.895

Values presented as mean ± standard deviation, quantity (%), or median (range). *p*-values from the Fisher exact test for categorical variables and Mann–Whitney U test for quantitative variables. PAVM, pulmonary arteriovenous malformation; FA, feeding artery; MVP, MicroVascular Plug; NiFA, nidus-plus-feeding-artery; USD, United States Dollar. * Only includes PAVMs with saccular morphology. † Calculated from 47 sessions with a single PAVM treated. ‡ Only includes PAVMs with long-term follow-up.

**Table 4:** Subgroup analysis of stratified durable occlusion treatment outcomes comparing the FA-MVP and NiFA-coil techniques during short-term follow-up (<18 months).

Feature	All PAVMS		FA-MVP Technique		NiFA Coil Technique		FA-MVP vs. NiFA Coil *p*-Value
Overall success	138/142 (97.2%)		47/48 (97.9%)		91/94 (96.8%)		1
Angioarchitecture							
Simple	102/103 (99.0%)	*p* = 0.063	44/45 (97.8%)	*p* = 1	58/58 (100%)	*p* = 0.054	0.437
Complex	36/39 (92.3%)		3/3 (100%)		33/36 (91.7%)		1
Nonsaccular	45/47 (95.7%)	*p* = 0.600	15/15 (100%)	*p* = 1	30/32 (93.8%)	*p* = 0.266	1
Saccular	93/95 (97.9%)		32/33 (96.7%)		61/62 (98.4%)		1
Feeding artery diameter							
Small (≤3 mm)	90/92 (97.8%)	*p* = 0.613	43/44 (97.2%)	*p* = 1	47/48 (97.9%)	*p* = 0.613	1
Large (>3 mm)	48/50 (96.0%)		4/4 (100%)		44/46 (95.7%)		1
Failure (mm)	3.9 ± 1.5	*p* = 0.283	2.5		4.3 ± 1.5	*p* = 0.381	0.500
Success (mm)	3.3 ± 1.8		2.3 ± 0.7		3.8 ± 1.9		<0.001
Sac diameter *							
Failure (mm)	10.1 ± 0.1	*p* = 0.736	10.1		10		1
Success (mm)	11.2 ± 7.9		7.6 ± 3.2		13.1 ± 8.9		<0.001

Values presented as mean ± standard deviation or quantity (%). *p*-values from the Fisher exact test for categorical variables and Mann–Whitney U test for quantitative variables. PAVM, pulmonary arteriovenous malformation; FA, feeding artery; MVP, MicroVascular Plug; NiFA, nidus-plus-feeding-artery. * Only includes PAVMs with saccular morphology.

**Table 5:** Subgroup analysis of stratified durable occlusion treatment outcomes comparing the FA-MVP and NiFA-Coil techniques during long-term follow-up (>3 years).

**Feature**	**All PAVMs**		**FA-MVP Technique**		**NiFA Coil Technique**		**FA-MVP vs. NiFA Coil** ***p*-Value**
Overall success	37/41 (90.2%)		13/14 (92.9%)		24/27 (88.9%)		1
Angioarchitecture							
Simple	29/30 (96.7%)	*p* = 0.052	12/13	*p* = 1	17/17 (100%)	*p* = 0.041	0.433
Complex	8/11 (72.7%)		1/1		7/10 (70%)		1
Nonsaccular	16/19 (84.2%)	*p* = 0.321	7/8 (87.5%)	*p* = 1	9/11 (81.8%)	*p* = 0.549	1
Saccular	21/22 (95.4%)		6/6 (100%)		15/16 (93.8%)		1
Feeding artery diameter							
Small (≤3 mm)	23/25 (92%)	*p* = 0.637	11/12 (91.7%)	*p* = 1	12/13 (92.3%)	*p* = 1	1
Large (>3 mm)	14/16 (87.5%)		2/2 (100%)		12/14 (85.7%)		1
Failure (mm)	3.9 ± 1.5	*p* = 0.214	2.5		4.3 ± 1.5	*p* = 0.410	0.500
Success (mm)	3.1 ± 1.7		2.1 ± 1.0		3.7 ± 1.8		0.004
Sac diameter *							
Failure (mm)	10.1		n/a		10.1		
Success (mm)	8.5 ± 6.2		4.2 ± 1.5		10.3 ± 6.5		0.004

Values presented as mean ± standard deviation or quantity (%). *p*-values from Fisher exact test for categorical variables and the Mann–Whitney U test for quantitative variables. PAVM, pulmonary arteriovenous malformation; FA, feeding artery; MVP, MicroVascular Plug; NiFA, nidus-plus-feeding-artery. * Only includes PAVMs with saccular morphology.

**Table 6:** Nidus-plus-feeding artery coil embolization treatment groups.

Embolic Group	Age *	Female *	HHT *	Follow-Up (Months)	Angioarchitecture		Feeding Artery Diameter (mm)	Embolic Quantity	Cost Analysis (USD)	Durable Occlusion
					**Simple**	**Complex**		**Number of Coils**		
Group 1 Non-HVDNF/DNFH Coils (n = 18)	40.6 ± 16.3	72.2%	77.8%	5.4 ± 4.4	59.3% (16/27)	40.7% (11/27)	4.0 ± 2.0 (2–11)	9.0 ± 6.4 (2–36)	6503 ± 4336 (1500–21.480)	96.3% (26/27)
Group 2 HVDNF/DNFH (n = 48)	46.9 ± 17.5	66.7%	81.3%	5.9 ± 4.1	62.7% (42/67)	37.3% (27/67)	3.4 ± 1.5 (2–11)	5.7 ± 4.5 (1–23)	7239 ± 5522 (1390–26.540)	97.0% (65/67)
Total	45.3 ± 17.5	66.7%	81.0%	5.8 ± 4.1	61.7% (58/94)	93.8% (45/48)	3.8 ± 1.9 (2–11)	6.7 ± 5.3 (1–36)	7027 ± 5197 (1390–26.540)	96.8% (91/94)
*p*-Value	1	0.772	0.739	0.485	0.817		0.138	<0.001	0.874	1

* Values are shown per patient rather than per PAVM (the remaining variables are presented per PAVM). HVDNF/DNFH, high-volume, detachable, non-fibered/detachable, non-fibered hydrogel.

**Table 7:** Comparison of pulmonary arteriovenous malformation (PAVM) Characteristics between FA-MVP and NiFA-Coil groups before and after propensity score weighting.

Characteristic	Raw	*p*-Value	Weighted
Feeding Artery Diameter (cm)	0.902	<0.001	0.315
PAVM Location	0.472	0.029	0.173
PAVM Angioarchitecture	0.715	<0.001	0.105

*p*-values from Fisher exact test for categorical variables and the Kruskal–Wallis rank sum test for quantitative variables. PAVM, pulmonary arteriovenous malformation; FA, feeding artery; MVP, MicroVascular Plug; NiFA, nidus-plus-feeding-artery.

**Table 8:** Hazards of persistence for NiFA-Coil with FA-MVP as the Referent.

Variable	Hazard Ratio	95% CI	*p*-Value
FA-MVP	1.00		
NiFA Coils	1.06	(0.16, 6.99)	0.956
Feeding artery diameter (cm)	1.24	(0, 434.97)	0.943

FA, feeding artery; MVP, MicroVascular Plug; NiFA, nidus-plus-feeding-artery. Inverse propensity score weighting was applied to the Cox proportional hazards regression model, with an adjustment for feeding artery diameter.

## Data Availability

Data contained within the article.
